# Pre-pregnancy underweight and obesity are positively associated with small-for-gestational-age infants in a Chinese population

**DOI:** 10.1038/s41598-019-52018-7

**Published:** 2019-10-29

**Authors:** Yuan Hua Chen, Li Li, Wei Chen, Zhi Bing Liu, Li Ma, Xing Xing Gao, Jia Liu He, Hua Wang, Mei Zhao, Yuan Yuan Yang, De Xiang Xu

**Affiliations:** 1 School of Basic Medical Sciences, Anhui Medical University, Hefei, 230032 China; 2 School of Public Health, Anhui Medical University, Hefei, 230032 China; 3 Department of Obstetrics and Gynecology, The First Affiliated Hospital of Anhui Medical University, Hefei, 230022 China; 4 Implantation and Placental Development Laboratory, Centre for Reproductive Health, Hudson Institute of Medical Research, Clayton, VIC Australia; 5 School of Nursing, Anhui Medical University, Hefei, 230032 China

**Keywords:** Epidemiology, Risk factors

## Abstract

The association between suboptimal pre-pregnancy body mass index (BMI) and small-for-gestational-age (SGA) infants is not well defined. We investigated the association between pre-pregnancy BMI and the risk of SGA infants in a Chinese population. We performed a cohort study among 12029 mothers with a pregnancy. This cohort consisted of pregnant women that were: normal-weight (62.02%), underweight (17.09%), overweight (17.77%) and obese (3.12%). Birth sizes were reduced in the underweight and obese groups compared with the normal-weight group. Linear regression analysis indicated that birth size was positively associated with BMI in both the underweight and normal-weight groups. Further analysis showed that 12.74% of neonates were SGA infants in the underweight group, higher than 7.43% of neonates reported in the normal-weight group (adjusted RR = 1.92; 95% CI: 1.61, 2.30). Unexpectedly, 17.60% of neonates were SGA infants in the obese group, much higher than the normal-weight group (adjusted RR = 2.17; 95% CI: 1.57, 3.00). Additionally, 18.40% of neonates were large-for-gestational-age (LGA) infants in the obese group, higher than 7.26% of neonates reported in the normal-weight group (adjusted RR = 3.00; 95% CI: 2.21, 4.06). These results suggest that pre-pregnancy underweight increases the risk of SGA infants, whereas obesity increases the risks of not only LGA infants, but also SGA infants.

## Introduction

Small-for-gestational-age (SGA) is one of the leading causes for stillbirth, neonatal deaths and perinatal morbidity^1–3^. A number of studies indicated that SGA was associated with diseases in childhood. An epidemiological study found that abnormal blood pressure in children born with SGA was more frequently observed than in children born with normal size^4^. Several studies demonstrated that children born with SGA had higher risks of developing diabetes mellitus, obesity and hyperlipidemia^5,6^. Moreover, a large retrospective cohort analysis showed that autism risk was increased in children born with preterm SGA^7^. On the other hand, SGA was associated with cardiovascular and metabolic diseases in adulthood. An earlier study found that serum IGF-I concentrations and the IGF-I/IGFBP-3 ratio were lower in adults that had SGA at birth, suggesting an association between SGA and an increased risk of metabolic diseases in adulthood^8^. Numerous epidemiological reports and animal experiments showed that adults from SGA pregnancy have higher blood pressure and cardio-metabolic risk than controls, suggesting an association between SGA and cardiovascular diseases in adulthood^9–11^.

The prevalence of suboptimal pre-pregnancy BMI has increased in recent years^12,13^. Previous studies investigating the association between pre-pregnancy BMI and the risk of SGA infants had inconsistent results. Several cohort studies showed that pre-pregnancy underweight increased the risk of SGA infants, whereas overweight and obesity were associated with a decreased risk of SGA infants^14–16^. Recently, a small sample of cohort study found that there was no significant association between pre-pregnancy BMI and the risk of SGA infants in a Chinese population^17^. Animal reports found that pre-pregnancy high fat diets-induced obesity decreased fetal weight and increased the incidence of SGA in mice^18,19^. Thus, whether suboptimal pre-pregnancy BMI influences the risk of SGA infants remains to be further determined in a large sample population.

In the present study, we performed a birth cohort study among 12029 mothers with a pregnancy. The aim of this study is to investigate the association between pre-pregnancy BMI and birth sizes. Additionally, we determine whether suboptimal pre-pregnancy BMI can influence the risk of SGA infants and large-for-gestational-age (LGA) infants.

## Results

### Demographic characteristics of study population

The demographic characteristics of pregnant women were presented in Table 1. According to pre-pregnancy BMI, 7461 pregnant women (62.02%) were normal-weight, 2056 (17.09%) underweight, 2137 (17.77%) overweight, and 375 (3.12%) obese. No subjects were drinking or smoking throughout the pregnancy. There were significant differences on maternal age, education, parity, gestational weight gain (GWG) and mode of delivery among different groups (Table 1). The incidence of pregnancy-induced hypertension, gestational diabetes mellitus and preeclampsia was significantly higher in the overweight and obese groups than those in the underweight and the normal-weight groups (Table 1). Moreover, there was a significant difference on gestational ages at the delivery among different groups (Table 1).

**Table 1:** Demographic characteristics of study population.

Characteristics	Pre-pregnancy BMI Category					*P* values
	Underweight		Normal-weight	Overweight	Obesity	
Pregnant women (n)	2056		7461	2137	375	
Maternal age [years, M ± SD]	27.4 ± 3.9		28.7 ± 4.4	30.3 ± 5.0	30.6 ± 5.1	<0.001
<25 [n (%)]	430 (20.91)		1134 (15.20)	224 (10.48)	43 (11.47)	<0.001
25–34 [n (%)]	1518 (73.84)		5543 (74.29)	1505 (70.43)	244 (65.07)	
≥35 [n (%)]	108 (5.25)		784 (10.51)	408 (19.09)	88 (23.46)	
**Maternal education (years)** ^**a**^						
Low [n (%)]	593 (28.84)		2255 (30.22)	814 (38.09)	194 (51.73)	<0.001
Medium [n (%)]	685 (33.32)		2334 (31.28	619 (28.96)	88 (23.47)	
High [n (%)]	732 (35.60)		2506 (33.59)	591 (27.66)	80 (21.33)	
Data missing [n (%)]	46 (2.24)		366 (4.91)	113 (5.29)	13 (3.47)	
**Mode of delivery**						
Natural delivery [n (%)]	1037 (50.44)		3466 (46.45)	747 (34.96)	86 (22.93)	<0.001
Cesarean delivery [n (%)]	1019 (49.56)		3995 (53.55)	1390 (65.04)	289 (77.07)	
Parity						
Nulliparous [n(%)]	1686 (82.00)		5805 (77.80)	1425 (66.68)	266 (70.93)	<0.001
Multiparous [n (%)]	370 (18.00)		1656 (22.20)	712 (33.32)	109 (29.07)	
**Gestational weight gain**						
Inadequate [n (%)]	428 (20.82)		1155 (15.48)	115 (5.38)	18 (4.80)	<0.001
Adequate [n (%)]	1073 (52.19)		3268 (43.80)	604 (28.26)	103 (27.47)	
Excessive [n (%)]	555 (26.99)		3038 (40.72)	1418 (66.35)	254 (67.73)	
**Pregnancy-induced hypertension** ^**b**^						
Yes [n (%)]	42 (2.04)		172 (2.31)	134 (6.27)	46 (12.27)	<0.001
No [n (%)]	2014 (97.96)		7289 (97.69)	2003 (93.73)	329 (87.73)	
**Gestational diabetes mellitus**						
Yes [n (%)]	72 (3.50)		526 (7.05)	318 (14.88)	89 (23.73)	<0.001
No [n (%)]	1984 (96.50)		6935 (92.95)	1819 (85.12)	286 (76.27)	
**Preeclampsia**						
Yes [n (%)]		77 (3.75)	364 (4.88)	206 (9.64)	66 (17.60)	<0.001
No [n (%)]		1979 (96.25)	7097 (95.12)	1931 (90.36)	309 (82.40)	
Gestational age (wks, M ± SD)		38.9 ± 2.0	38.8 ± 2.5	38.5 ± 2.7	37.8 ± 3.1	<0.001
**Infant sex**						
Boys [n (%)]		1084 (52.72)	3942 (52.83)	1188 (55.59)	188 (50.13)	0.075
Girls [n (%)]		972 (47.28)	3519 (47.17)	949 (44.41)	187 (49.87)	

Abbreviations: n, number; M, mean; SD, standard deviation.

^a^Low (junior school or less), medium (high school), high (College or above).

^b^Seventy-five pregnant women suffered from both pregnancy-induced hypertension and preeclampsia.

The mean differences among different groups were analyzed using one-way ANOVA. Categorical variables were analyzed using χ2 tests.

### Birth sizes among different groups

The correlations between pre-pregnancy BMI and birth sizes were analyzed. There were no significant correlations between pre-pregnancy BMI and birth weight (r = 0.059), birth length (r = 0.005), head circumference (r = 0.068) and chest circumference (r = 0.060). Birth sizes were compared among four groups. Birth sizes, including birth weight, birth length, head circumference and chest circumference, were significantly lower in the underweight and the obese groups than those in the normal-weight group (Table 2). Birth weight, head circumference and chest circumference were higher in the overweight group than those in the normal-weight group (Table 2).

**Table 2:** Birth sizes among different groups.

Parameter	Pre-pregnancy BMI Category			
	Underweight	Normal-weight	Overweight	Obesity
**Birth weight (g)**				
Mean ± SD^a^	3072.3 ± 550.4**	3181.7 ± 608.7	3245.5 ± 713.6**	3068.4 ± 897.3**
Median (10^th^, 25^th^, 75^th^, 90^th^)^b^	3100 (2400, 2800, 3400, 3700)**	3250 (2400, 2900, 3550, 3850)	3350 (2200, 2950, 3700, 4000)**	3250 (1620, 2550, 3750, 4050)
**Birth length (cm)**				
Mean ± SD^a^	49.4 ± 2.9**	49.8 ± 3.2	49.8 ± 3.5	48.8 ± 4.6**
Median (10^th^, 25^th^, 75^th^, 90^th^)^b^	50.0 (46.0, 48.0, 51.0, 52.0)**	50.0 (46.0, 49.0, 52.0, 53.0)	50.0 (46.0, 49.0, 52.0, 53.0)	50.0 (42.0, 47.0, 52.0, 53.0)**
**Head circumference (cm)**				
Mean ± SD^a^	33.0 ± 2.0**	33.3 ± 2.2	33.6 ± 2.3**	33.0 ± 3.2**
Median (10^th^, 25^th^, 75^th^, 90^th^)^b^	33.0 (31.0, 32.0, 34.0, 35.0)**	33.5 (31.0, 32.0, 35.0, 36.0)	34.0 (31.0, 32.0, 35.0, 36.0)**	34.0 (28.0, 32.0, 35.0, 37.0)
**Chest circumference (cm)**				
Mean ± SD^a^	32.6 ± 2.3**	33.0 ± 2.5	33.3 ± 2.8**	32.6 ± 3.6**
Median (10^th^, 25^th^, 75^th^, 90^th^)^b^	33.0 (30.0, 32.0, 34.0, 35.0)**	33.0 (30.0, 32.0, 34.0, 36.0)	34.0 (30.0, 32.0, 35.0, 36.0)**	33.0 (27.0, 31.0, 35.0, 36.0)

^a^The mean differences between two groups were analyzed using least significant difference (LSD) post hoc test.

^b^The median differences were analyzed using non-parametric statistics.

**P < 0.01 as compared with normal-weight.

### Association between pre-pregnancy BMI as a categorical variable and the risks of SGA infants

The association between pre-pregnancy BMI and the risk of SGA infants was analyzed. As shown in Table 3, 12.74% of SGA infants were from the underweight group and 17.60% from the obese group, significantly higher than 7.43% of SGA infants from the normal-weight group. After adjustment for confounders, results showed that not only underweight but also obesity increased the risk of SGA infants (Table 3). However, overweight was not associated with an increased risk of SGA infants (Table 3).

**Table 3:** Crude and adjusted RRs for the association between BMI and SGA.

Parameter	Pre-pregnancy BMI Category			
	Underweight	Normal-weight	Overweight	Obesity
SGA [n (%)]	262 (12.74)	554 (7.43)	146 (6.83)	66 (17.60)
Crude *RR* (95% *CI*)	1.82 (1.56, 2.13)**	1.00	0.91 (0.76, 1.10)	2.66 (2.01, 3.52)**
Adjusted *RR* (95% *CI*)^a^	1.79 (1.53, 2.10)**	1.00	0.93 (0.77, 1.13)	2.73 (2.06, 3.61)**
Adjusted *RR* (95% *CI*)^b^	1.83 (1.55, 2.16)**	1.00	0.84 (0.69, 1.03)	2.25 (1.67, 3.02)**
Adjusted *RR* (95% *CI*)^c^	1.91 (1.61, 2.26)**	1.00	0.96 (0.78, 1.17)	2.51 (1.85, 3.41)**
Adjusted *RR* (95% *CI*)^d^	1.92 (1.61, 2.30)**	1.00	0.92 (0.74, 1.14)	2.17 (1.57, 3.00)**

Abbreviations: BMI, body mass index; SGA, small-for-gestational-age.

^a^Adjustment for maternal age.

^b^Adjustment for parity and maternal education.

^c^Adjustment for gestational weight gain.

^d^Adjustment for maternal age, gestational weight gain, parity and maternal education.

Multiple logistic regression models were used to calculate crude and adjusted RR with 95% CI. **P < 0.01 as compared with normal-weight.

### Correlation between pre-pregnancy BMI as a continuous variable and birth sizes

Linear regression was then used to explore the correlation between pre-pregnancy BMI and birth sizes. Crude linear regression analyses showed that pre-pregnancy BMI was positively associated with birth weight, birth length and chest circumference among underweight women (Table 4). Pre-pregnancy BMI was associated with birth weight, birth length, head and chest circumference among normal-weight women (Table 4). After adjustment for maternal age, GWG, parity and maternal education, pre-pregnancy BMI was positively associated with birth weight, birth length, head and chest circumference among both underweight and normal-weight women (Table 4).

**Table 4:** Association between maternal pre-pregnancy BMI and birth sizes based on linear regression analyses.

	Birth weight (g)		Birth length (cm)		Head circumference (cm)		Chest circumference (cm)	
	β (95% CI)	*p*	β (95% CI)	*p*	β (95% CI)	*p*	β (95% CI)	*p*
**Crude models**								
All	11.7 (7.9, 15.5)	<0.001	0.00 (−0.02, 0.02)	0.964	0.05 (0.04, 0.06)	<0.001	0.05 (0.03, 0.07)	<0.001
Underweight	58.4 (28.5, 88.3)	<0.001	0.20 (0.05, 0.36)	0.012	0.09 (−0.04, 0.15)	0.105	0.21 (0.09, 0.34)	0.001
Normal-weight	30.2 (18.8, 41.6)	<0.001	0.11 (0.05, 0.17)	<0.001	0.13 (0.09, 0.17)	<0.001	0.14 (0.09, 0.19)	<0.001
Overweight	1.1 (−24.6, 26.8)	0.934	−0.05 (−0.16, 0.07)	0.487	0.00 (−0.08, 0.09)	0.976	−0.00 (−0.10, 0.10)	0.980
Obesity	−15.9 (−45.7, 13.9)	0.296	0.03 (−0.11, 0.17)	0.664	−0.05 (−0.16, 0.06)	0.360	−0.02 (−0.13, 0.10)	0.794
**Adjusted models** ^**a**^								
All	16.8 (12.6, 20.9)	<0.001	0.02 (0.00, 0.04)	0.039	0.05 (0.03, 0.06)	<0.001	0.07 (0.05, 0.08)	<0.001
Underweight	57.9 (27.1, 88.7)	<0.001	0.18 (0.02, 0.34)	0.026	0.08 (−0.03, 0.20)	0.162	0.20 (0.07, 0.32)	0.002
Normal-weight	42.3 (30.3, 54.4)	<0.001	0.18 (0.12, 0.24)	<0.001	0.14 (0.10, 0.19)	<0.001	0.18 (0.13, 0.23)	<0.001
Overweight	12.4 (−15.7, 40.5)	0.387	0.01 (−0.15, 0.13)	0.861	0.02 (−0.08, 0.11)	0.712	0.02 (−0.09, 0.13)	0.697
Obesity	−17.9 (−48.1, 12.2)	0.243	0.01 (−0.15, 0.17)	0.859	−0.09 (−0.20, 0.03)	0.138	−0.05 (−0.18, 0.07)	0.389

^a^Adjustment for maternal age, gestational weight gain, parity and maternal education.

Linear regression was used to explore the association between pre-pregnancy BMI and birth sizes.

### Association between pre-pregnancy BMI as a categorical variable and the risk of LGA infants

The association between pre-pregnancy BMI and the risk of LGA infants was analyzed using multiple logistic regression analysis. As shown in Tables 5, 4.09% of neonates were LGA infants from the underweight group, significantly lower than 7.26% reported from the normal-weight group (RR = 0.54). Additionally, 13.43% of neonates were LGA infants from the overweight group (RR = 1.91) and 18.40% from the obese group (RR = 2.88), higher than the normal-weight group (Table 5). After adjustment for different confounder, results showed that not only overweight but also obesity increased the risk of LGA infants (Table 5). Underweight was associated with a decreased risk of LGA infants (Table 5).

**Table 5:** Crude and adjusted RRs for the association between BMI and LGA.

Parameter	Maternal pre-pregnancy BMI			
	Underweight	Normal-weight	Overweight	Obesity
LGA [n (%)]	84 (4.09)	542 (7.26)	287 (13.43)	69 (18.40)
Crude *RR* (95% *CI*)	0.54 (0.43, 0.69)**	1.00	1.91 (1.64, 2.23)**	2.88 (2.19, 3.79)**
Adjusted *RR* (95% *CI*)^a^	0.55 (0.43, 0.69)**	1.00	1.89 (1.62, 2.21)**	2.85 (2.16, 3.76)**
Adjusted *RR* (95% *CI*)^b^	0.57 (0.45, 0.72)**	1.00	2.00 (1.70, 2.36)**	3.02 (2.24, 4.06)**
Adjusted *RR* (95% *CI*)^c^	0.64 (0.50, 0.82)**	1.00	1.43 (1.21, 1.68)**	2.15 (1.61, 2.87)**
Adjusted *RR* (95% *CI*)^d^	0.56 (0.43, 0.71)**	1.00	1.98 (1.67, 2.34)**	3.00 (2.21, 4.06)**

Abbreviations: BMI, body mass index; SGA, small-for-gestational-age.

^a^Adjustment for maternal age.

^b^Adjustment for parity and maternal education.

^c^Adjustment for gestational weight gain.

^d^Adjustment for maternal age, gestational weight gain, parity and maternal education.

Multiple logistic regression models were used to calculate crude and adjusted RR with 95% CI. **P < 0.01 as compared with normal-weight.

### Demographic characteristics between women who had SGA or LGA infants within the obese category

As shown in Table 6, no significant differences were observed on pre-pregnancy BMI, pregnancy-induced hypertension and preeclampsia between the SGA group and the LGA group within the obese category. However, there were significant differences on GWG, advanced maternal age and low education between the SGA group and the LGA group (Table 6). The incidence of gestational diabetes mellitus was significantly higher in the LGA group than that in the SGA group (Table 6).

**Table 6:** Maternal characteristics between women who had SGA and LGA infants within the obese category.

Characteristics	SGA	LGA	*P* values
Pregnant women (n)	66	69	
Maternal age (years, M ± SD)	31.8 ± 5.4	29.8 ± 5.0	0.022
<25 [n (%)]	4 (6.06)	8 (11.59)	0.039
25–34 [n (%)]	37 (56.06)	48 (69.57)	
≥35 [n (%)]	25 (37.88)	13 (18.84)	
Pre-pregnancy BMI (kg/m^2^, M ± SD)	30.58 ± 2.80	30.67 ± 3.53	0.873
**Maternal education (years)** ^**a**^			
Low [n (%)]	46 (69.70)	30 (43.48)	0.005
Medium [n (%)]	13 (19.70)	19 (27.54)	
High [n (%)]	7 (10.61)	20 (28.99)	
**Gestational weight gain**			
Inadequate [n (%)]	11 (16.67)	0 (0.00)	<0.001
Adequate [n (%)]	24 (36.36)	3 (4.35)	
Excessive [n (%)]	31 (46.97)	66 (95.65)	
**Pregnancy-induced hypertension**			
Yes [n (%)]	2 (3.03)	8 (11.59)	0.097
No [n (%)]	64 (96.97)	61 (88.41)	
**Gestational diabetes mellitus**			
Yes [n (%)]	7 (10.61)	29 (42.03)	<0.001
No [n (%)]	59 (89.39)	40 (57.97)	
**Preeclampsia**			
Yes [n (%)]	11 (16.67)	10 (14.49)	0.814
No [n (%)]	55 (83.33)	59 (85.51)	

^a^Low (junior school or less), medium (high school), high (College or above).

The mean differences between two groups were analyzed using least significant difference (LSD) post hoc test. Categorical variables were analyzed using χ2 tests.

## Discussion

Lower birth size may have a major impact on the risk of adult diseases. Several cohort studies showed that people who had lower birth sizes were at increased risks of developing cardiovascular diseases including stroke, higher systolic blood pressure, and coronary heart disease^20,21^. Evidence from animal experiments and epidemiological studies demonstrated that lower birth sizes were associated with metabolic disorders including higher BMI and diabetes in adulthood^22,23^. The present study analyzed the association between pre-pregnancy BMI and birth sizes including birth weight, birth length, head circumference and chest circumference in a birth cohort study that included 12029 mothers with a pregnancy. Results showed positive correlations between pre-pregnancy BMI and birth sizes.

Maternal demographic characteristics, such as maternal age, GWG and maternal education, were important confounding variables for relationships to birth sizes and the risk of SGA infants. A number of epidemiological studies demonstrated that advanced maternal age increased the risk of SGA infants^24^. A recent meta-analysis demonstrated that excessive GWG was associated with a decreased risk of SGA infants among underweight and normal-weight women, and an increased risk of LGA infants among normal-weight, overweight and obese women. In contrast, Inadequate GWG was associated with an increased risk of SGA infants among underweight and normal-weight women but not among overweight and obese women^25^. Low educational subjects had higher risk of SGA infants as compared with high educational subjects^26,27^. Few studies have taken into account the effect of these confounders on the association between pre-pregnancy BMI and SGA infants. In the present study, there were significant differences on maternal age, education, GWG and mode of delivery among different groups. Thus, adjusted RRs with 95% CI were estimated in the present study. After adjustment for these confounders, our results showed that not only underweight but also obesity was associated with an increased risk of SGA infants.

The present study showed that maternal pre-pregnancy underweight was associated with an increased risk of SGA infants, consistent with previous research works^14–16^. Moreover, the present study found that pre-pregnancy obesity increased the risks of not only LGA infants but also SGA infants. In contrast, several cohort studies showed that obesity was associated with an increased risk of LGA infants and a decreased risk of SGA infants^14–16^. A recent cohort study showed that there was no association between pre-pregnancy BMI and the risk of SGA infants in a Chinese population^17^. The inconsistency of early reports may be related to several reasons: firstly, most early reports were implemented in developed countries or Euro-American countries^14–16^. There are significant differences on body structure, BMI classifications, race and ethnicity between these populations and Chinese population^14–16,28^. In fact, several studies showed that pre-pregnancy BMI was associated with birth weight among European Americans but not African Americans, suggesting a racial/ethnic difference on the relationship between pre-pregnancy BMI and fetal growth^29,30^; secondly, negative results came most frequently from small samples^17^; Lastly, animal studies demonstrated that pre-pregnancy and/or gestational high fat diets (HFD)-induced obesity differentially disturbed fetal growth development^18,19,31^. Pre-pregnancy HFD-induced obesity caused fetal SGA. By contrary, gestational HFD-induced obesity leaded to fetal overweight. To our knowledge, the present study demonstrates that obesity increases the risk of not only LGA infants but also SGA infants in a large sample of population.

The mechanism by which obesity increased the risk of SGA infants remains unclear. Other studies showed that the levels of pro-inflammatory cytokines and chemokines in placenta and maternal serum were significantly higher among obese pregnant women compared to controls^32,33^. Animal studies demonstrated that HFD-induced obesity could induce low-grade systemic inflammation through activating nuclear factor (NF)-κB pathway and interferon regulatory factor (IRF)-3 signaling^18,34^. Indeed, several epidemiological reports demonstrated that the levels of tumor necrosis factor (TNF)-α, C-reactive protein and interleukin (IL)-8 were significantly higher in maternal serum and umbilical cord serum of SGA infants than in controls^35,36^. Furthermore, maternal inflammation during pregnancy impaired fetal development by disturbing placental spiral artery remodeling and nutrient transport capacity^37–39^. These results suggest that placental inflammation may play a vital role in obesity-mediated SGA infants. Moreover, pre-pregnancy obesity was associated with elevation of placenta weight and up-regulation of placental nutrient transporters^40,41^. Animal studies showed that placental transporters for glucose, fatty acids and amino acids were significantly up-regulated in obese mice^42,43^. Therefore, we speculate that pre-pregnancy obesity-induced LGA infants may be attributing to placenta overgrowth and up-regulation of placental nutrient transporters.

The current study also compared maternal demographic characteristics between women who had SGA vs LGA infants within the obese category. There were no significant differences on pre-pregnancy BMI, pregnancy-induced hypertension and preeclampsia between the SGA group and the LGA group within the obese category. However, there were significant differences on GWG, advanced maternal age, low education and gestational diabetes mellitus between the SGA group and the LGA group. These results suggest that inadequate GWG, advanced maternal age and low education might be associated with an increased risk of SGA infants among obese category. Therefore, gestational diabetes mellitus and excessive GWG may be associated with an increased risk of LGA infants within the obese category. Nevertheless, more tests are required to investigate the underlying mechanisms through which obesity can lead to the different birth outcomes.

In summary, our results showed that birth sizes were positively associated with pre-pregnancy BMI in both the underweight and normal-weight groups. We also found that pre-pregnancy underweight increased the risk of SGA infants, whereas obesity increased the risk of not only LGA infants, but also SGA infants.

## Subjects and Methods

### Cohort study

We performed a birth cohort study that included 13801 pregnant women between January 2011 and December 2014 attended The First Affiliated Hospital of Anhui Medical University for their antenatal care and delivery in Hefei, China. Eight hundred and ninety-seven pregnant women no detailed delivery records, 270 fetal deaths or stillbirths, 294 pregnant women giving birth to multiple births, 147 induced-abortions and 164 unavailable pre-pregnancy BMI data were excluded from this study. Finally, 12029 (87.2%) mothers with a pregnancy were recruited for this study. Pre-pregnancy BMI was categorized according to the WHO cut-points for Asian adults: BMI < 18.5 kg/m^2^ for underweight, 18.5 kg/m^2^ ≤ BMI < 23 kg/m^2^ for normal-weight, 23 kg/m^2^ ≤ BMI < 27.5 kg/m^2^ for overweight and BMI ≥ 27.5 kg/m^2^ for obesity^44,45^. According to the 2009 Institute of Medicine (IOM) recommendations, gestational weight gain (GWG) was categorized as follow^46^. Inadequate: GWG <12.5 kg in underweight women, <11.5 kg in normal-weight women, <7 kg in overweight women, and <5 kg in obese women. Adequate: 12.5 ≤ GWG ≤ 18 kg in underweight women, 11.5 ≤ GWG ≤ 16 kg in normal-weight women, 7 ≤ GWG ≤ 11.5 kg in overweight women, and 5 ≤ GWG ≤ 9 kg in obese women. Excessive: GWG > 18 kg in underweight women, >16 kg in normal-weight women, >11.5 kg in overweight women, and >9 kg in obese women. The present study obtained ethics approval from the ethics committee of Anhui Medical University (No. 20160010). All participants signed the written informed consent form for this study. All methods were carried out in accordance with the approved ethic guidelines.

### Definition of pregnancy-induced hypertension and preeclampsia

Pregnancy-induced hypertension was defined by a systolic blood pressure (BP) 140 mmHg and/or diastolic BP 90 mmHg, based on the average of at least two measurements, taken at least 15 min apart, using the same arm^47^. Preeclampsia was defined by new-onset proteinuria and potentially, other end-organ dysfunction.

### Definition of small-for-gestational age and large-for-gestational age

Small-for-gestational age (SGA) and large-for-gestational age (LGA) were designed as birth weight of live-born infants <10^th^ percentile and å 90^th^ percentile based on gender and gestational age from a reference population for Chinese, respectively^48^.

### Statistical analysis

SPSS 17.0 was used to analysis the data. The mean differences were analyzed using one-way ANOVA and least significant difference (LSD) post hoc test. Categorical variables were analyzed using χ2 tests. The median differences were analyzed using non-parametric statistics (Mann-Whitney U test). Linear regression was used to explore the association between pre-pregnancy BMI and birth sizes. Multiple logistic regression models were used to calculate crude and adjusted relative risk (RR) with 95% confidence intervals (95% CI) with respect to SGA infants. A *p*-value of < 0.05 (two-tailed) or a 95% CI not including 1 was considered statistically significant.
